# Reconstruction of the labia majora using a neurovascular pedicled pudendal thigh flap after extensive vulvectomy for primary extramammary Paget's disease: A case report

**DOI:** 10.1002/ccr3.7660

**Published:** 2023-07-17

**Authors:** Jiangbo Cui, Yu Zhang, Zhou Yu, Xing Fan, Xi Zhang, Li Yang, Baoqiang Song, Yang Li

**Affiliations:** ^1^ Department of Plastic Surgery Xijing Hospital Fourth Military Medical University Shaanxi China

**Keywords:** labia defect, pedicled pudendal thigh flap, primary extramammary Paget's disease, reconstruction, wound healing

## Abstract

**Key Clinical Message:**

A neurovascular pedicled pudendal thigh flap was used to reconstruct labial defect after extensive vulvectomy for primary extramammary Paget's disease. The flap was reliable for the superior skin and ideal for large labial defect reconstruction.

**Abstract:**

Extramammary Paget's disease (EMPD), a rare type of intraepidermal carcinoma, predominantly affects the genitalia. Generally, the treatment for primary EMPD is wide surgical excision; however, large tissue defects after resection necessitate customized reconstruction. Previously, several reconstruction techniques have been outlined, such as local skin flaps (V‐Y flaps), skin grafts, pedicled flaps, and free flaps. However, the complexity of EMPD management is due to the multiple flaps and techniques. In this case report, we applied a neurovascular pedicled pudendal thigh flap to reconstruct a labial defect in a 68‐year‐old woman using the technique of elevation and particularly the insetting of the flap. This pedicled flap was robust and reliable, producing a labium that was natural in appearance with good‐quality skin cover and a protective sensation. The patient was satisfied with both the cosmetic appearance and normal sensation of the reconstructed labia. Additionally, the linear scar at the donor site was located along the inguinal fold, and the flap was accepted by the patient as an ideal tissue for reconstruction of the large labial defect.

## Graphical abstract

Posterior neurovascular pedicled pudendal thigh flaps was applied for the treatment of Extramammary Paget's disease. The flap is based on the branches of the internal pudendal artery, which originate from the internal iliac artery.

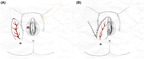

## 1 INTRODUCTION

Extramammary Paget's disease (EMPD), a rare intraepidermal carcinoma, primarily affects the genitalia region.^1^, ^2^ It is classified as primary and secondary EMPD. They are distinct from whether there is an existence of an underlying malignancy such as adenocarcinoma originated from skin appendages or non‐cutaneous carcinoma.

Less than 1% of vulva neoplasms are attributed to EMPD of the labia,^3^ and the majority of primary lesions are limited to the epidermis. Wide and local excision is a suitable treatment; but it generates a large secondary wound defect. Skin grafts have been mainly used for labial reconstruction. Besides, application of myocutaneous flaps also has been described. These techniques have advantages and limitations. Indeed, the skin grafts are particularly effective for treating superficial defects; however, the application is restricted in cases of major resections. Due to the limited tissue supply, the use of local skin flaps (V‐Y flaps) is restricted in patients with complete agenesis. The most disturbing disadvantage of musculocutaneous flaps, including those of the gracilis^4^ and rectus abdominis,^5^ is that they cause bulky tissue formation, with additional disadvantages such as conspicuous donor‐site scars or additional potential for morbidity and disfigurement. Therefore, the techniques used for the closure of a large wound defect after labial excision must be tailored to the wound geometry and potential secondary complications must be considered.

**FIGURE 1:**
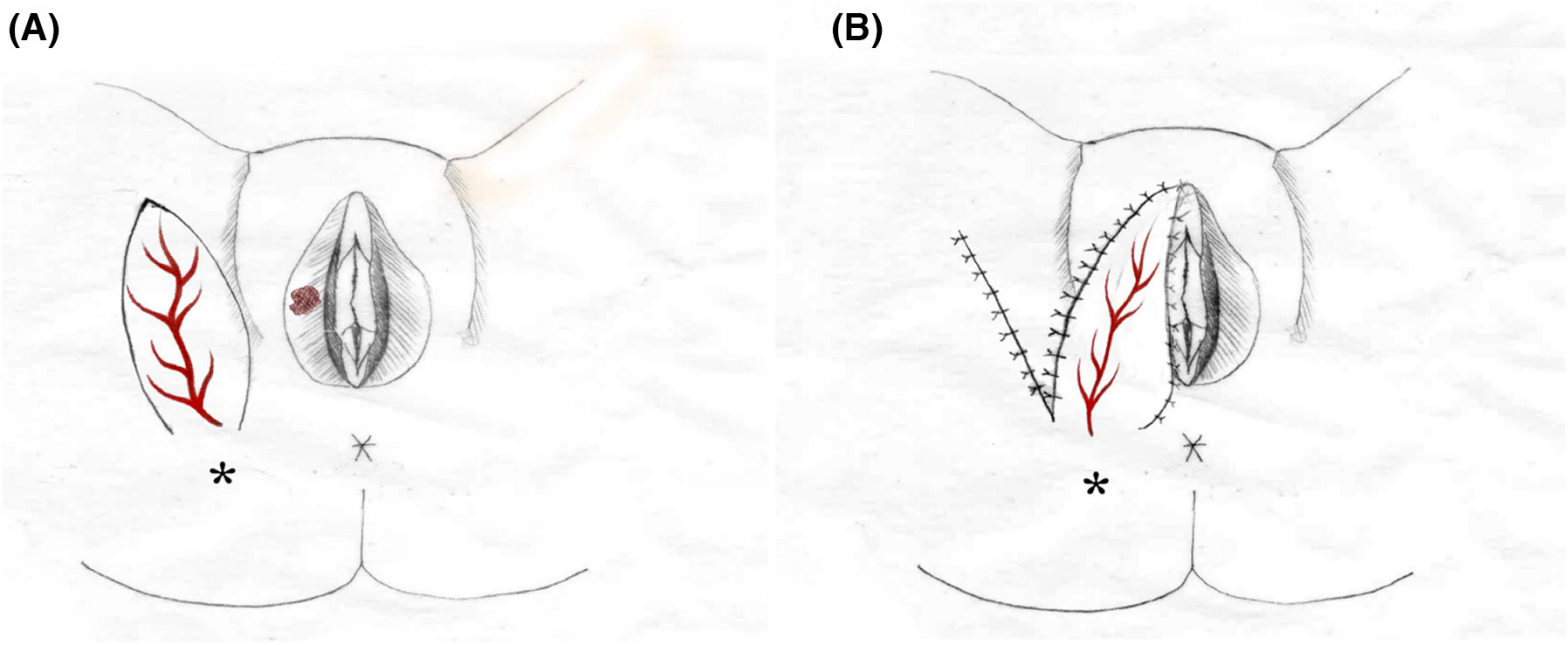
Schematic drawing of the neurovascular pedicled pudendal thigh flap. (A) Ischial tuberosity. The red lines represent the branch of the pudendal artery that supplies the skin, which was identified using Doppler ultrasonography before the procedure. (B) A postoperative view of the transposed flap is shown.

Posterior neurovascular pedicled pudendal thigh flaps for vaginal reconstruction were first described by Wee and Joseph more than two decades ago.^6^ Reports have indicated positive effects with regard to patient tolerance, flap viability, and donor‐site morbidity.^7^, ^8^ The pudendal thigh flap is based on the branches of the internal pudendal artery, which originate from the internal iliac artery (Figure 1).^9^ In addition, this flap is innervated by the posterior labial branches of the pudendal nerve and the perineal rami of the posterior cutaneous nerve of the thigh.^10^ This flap is suitable for labial defect reconstruction as it provides sensation and has minimal donor area morbidity. In this report, a method for reconstructing large labial defects using a neurovascular pedicled pudendal thigh flap based on a cutaneous branch of the internal pudendal artery is described.

### 1.1 Patient

**FIGURE 2:**
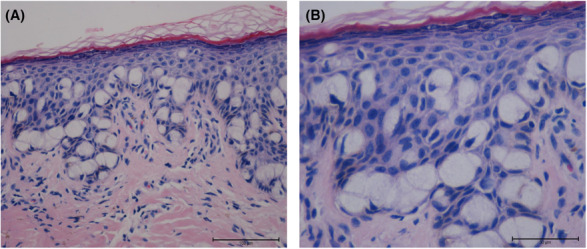
Tissue section image of primary Paget's disease of the labia. Hematoxylin–eosin (HE) staining of primary Paget's disease of the labia. Paget cells with large nuclei, prominent nucleoli, and pale cytoplasm extend into the basal and parabasal areas of the epithelium. (A) HE ×200; (B) HE ×400.

**FIGURE 3:**
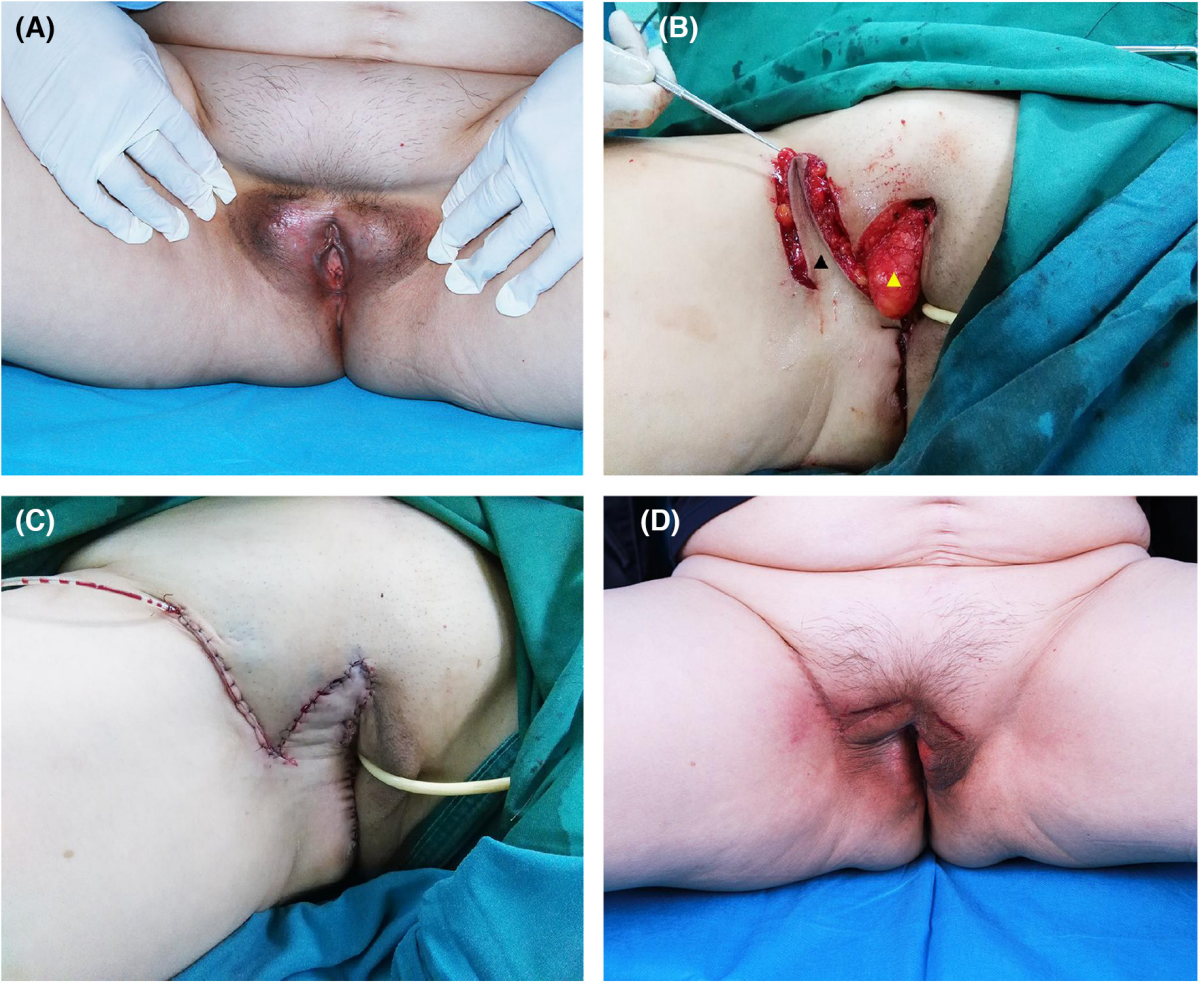
The 68‐year‐old woman with Paget's disease of the right labia who underwent a reconstruction with a neurovascular pedicled pudendal thigh flap. (A) The right labial lesion is shown preoperatively. (B) The labia defect is shown intraoperatively after the tumor resection (▲) and harvesting of the flap. The design of the flap in the right groin crease region that included cutaneous branches of pudendal artery is shown (▲). (C) The flap is translocated to the defect, and the donor site is closed along the inguinal crease. (D) The flap is shown 6 months postoperatively.

A 68‐year‐old woman complained of minor pain and redness in the genital region for 5 years. Outpatient treatment at an outside facility had been unsuccessful. The patient was referred to our outpatient department for further assessment. A biopsy of the lesion tissue on the right labia majora was conducted, and histopathological images showed numerous cells with abundant, pale cytoplasm; large, pleomorphic nuclei; and prominent nucleoli. The nuclei were eccentric and resembled signet rings (Figure 2). The size of tumor was about 2.5 × 0.8 cm (Figure 3A). Therefore, primary Paget's disease of the labia was diagnosed. No further investigations revealed distal metastasis, and a cystoscopic examination showed no other defects in the bladder.

### 1.2 Flap design and surgical technique

A radical vulvectomy, with a labial tissue defect (10.5 × 6.5 cm, Figure 3B), was performed while the patient was in the prone position. Surgical resection included a 3‐cm margin around the lesion. Complete removal of the lesion was confirmed using frozen sections obtained during the procedure. At the time of the excision, primary wound closure was not suitable, so the main goal was the transposition of well‐vascularized tissue with sufficient volume to fill the defect and reconstruct the labia majora. Consequently, a unilateral pudendal thigh flap was designed with a groin crease localized in the midline of the posteriorly‐based flap to hide the scar. Using a portable Doppler ultrasonography device, the location of the internal pudendal artery along the right gluteal fold was identified. The size of the designed flap (12.0 × 6.0 cm) was proportional to the size of the defect. The flap was elevated under the deep fascia to include the posterior pudendal vessels and nerves to ensure the sensation and survivability of the flap (Figure 3C). After transposing the flap to repair the defect without tension, the donor‐site wound was closed with minimal subcutaneous undermining. The shape and texture of the labia were satisfactory. Negative suction drainage was inserted immediately and removed on postoperative Day 3. The total operating time was 118 min. This study has been approved by the ethics committee review board, and the patient provided consent for the publication of this report.

## 2 RESULTS

The flap remained viable, and no signs of tissue necrosis were observed after the repair. The successful preservation of sensation was verified in all areas of the flap immediately after the surgery. After 1 month, the wound healed without complications. At the 6‐month follow‐up, favorable aesthetic results were noted (Figure 3D).

## 3 DISCUSSION

EMPD is similar to Paget's disease of the breast in histological images.^11^ It is an epidermal neoplasia that is difficult to treat and accounts for <1% of all labial neoplasms. EMPD predominantly occurs in the fifth and eighth decades of life, with an average age of onset of 70 years.^12^ It primarily affects the genital area, including the labia, penis, scrotum, and perianal regions.

Local excision is an effective method for treating EMPD. For intraepithelial lesions, due to the multicentric nature of the disease and frequent microscopic extensions beyond the visible boundaries of the tumor, wide excision that covers a margin of 2–3 cm may usually be performed. Wide local excision using the Moh's microscopic technique or intraoperative frozen sections may reduce the rate of positive margins and rate of recurrence once confirmed via biopsy. However, a complete excision results in a large defect that requires reconstruction using primary closure, skin grafts, or distant flap transplantations. These methods are related to unfavorable long‐term results due to secondary infection, long‐term confinement in the prone position to immobilize the skin graft, and perineal malformations caused by graft contracture. Skin flap reconstruction is clinically indicated for functional and cosmetic preservation.^13^

A case of non‐invasive, primary EMPD located in the right labia majora was presented in this report. As the tumor was large, a wide excision of the lesion with a 3‐cm safety margin was conducted. The resulting labial defect was too large to repair using a local flap. Furthermore, the patient desired cosmetic preservation in addition to surgical repair, rendering the reconstruction more challenging. Vertical or transverse rectus abdominis or gracilis flaps, which are myocutaneous flaps, are often unsuitable for labial reconstruction due to their excessive bulkiness, leading to further risks of donor‐site morbidity and disfigurement.^14^ In this patient, a neurovascular pedicled pudendal thigh flap was used as a fasciocutaneous flap, offering a labial coverage with a more anatomically appropriate thickness compared to other flaps.

In addition, the posterior pudendal vessels and nerves in this flap provide excellent blood supply and sensation. Therefore, reconstruction using a neurovascular pedicled pudendal thigh flap is more advantageous than other surgical approaches for repairing large labial defects due to EMPD.

## 4 CONCLUSIONS

Vulvar defects can result from several types of insults. Injuries and tumor excision may present challenges for the clinical management and reconstruction of the defects. Skin tissue flaps containing the internal pudendal artery provide ideal materials for tension‐free, anatomical reconstruction of extended perineal defects. Moreover, the absence of thigh donor‐site deformities and scarring, as well as the simplified labia majora restoration, could potentially expand its indications in the future.

## AUTHOR CONTRIBUTIONS

**Jiangbo Cui:** Conceptualization; data curation; formal analysis; investigation; methodology. **Yu Zhang:** Conceptualization; data curation; formal analysis; investigation; methodology. **Zhou Yu:** Data curation; formal analysis; methodology. **Xing Fan:** Resources. **Xi Zhang:** Resources. **Li Yang:** Resources. **Baoqiang Song:** Conceptualization; methodology; project administration; resources; supervision. **Yang Li:** Conceptualization; funding acquisition; methodology; project administration; resources; supervision; writing – review and editing.

## CONFLICT OF INTEREST STATEMENT

The authors declare no conflicts of interest.

## CONSENT

Written informed consent was obtained from the patient to publish this report in accordance with the journal's patient consent policy.

## Data Availability

The authors confirm that the data supporting the findings of this study are available within the article.
